# Massage of a Hematoma to Assist in Decreasing the Volume of an Intraparenchymal Hemorrhage

**DOI:** 10.7759/cureus.12227

**Published:** 2020-12-22

**Authors:** Zhenjiang Pan, Jing Bao, Shepeng Wei

**Affiliations:** 1 Neurosurgery, Shidong Hospital of Yangpu District in Shanghai, Shanghai, CHN

**Keywords:** massage, spontaneous intracerebral hemorrhag, ich, minimally invasive surgery

## Abstract

Spontaneous intracerebral hemorrhage (ICH) is one of the least treatable types of stroke, and its incidence and all-age mortality have increased over the last several decades in China. Surgical evacuation using standard craniectomy for supratentorial hematoma is always controversial. How to ensure effective decreasing of intracranial pressure (ICP) is crucial to the management of ICH. A 48-year-old right-handed woman was brought to our hospital by her family, who stated that the woman could not speak well and had developed sudden left-sided weakness three hours previously. The patients were diagnosed with supratentorial bilateral intraparenchymal hemorrhages, mainly in the putaminal area. After inserting a catheter into the hematoma, we began to perform the maneuver of massage through the catheter by frequently using multiple 2 mL quantities of normal saline and performing the injecting-aspiration maneuver. After performing this massage repeatedly for 15 min, we terminated the operation. After the patient was admitted to the ICU, urokinase (40,000 U) was administered through the catheter every 12 hours for three days. After the patient stayed for an additional 11 days, she was discharged home. Minimally invasive surgery with massage techniques followed by thrombolytic evacuation of clots will be a new method for treating ICH patients.

## Introduction

Spontaneous intracerebral hemorrhage (ICH) is one of the least treatable types of stroke, and its incidence and all-age mortality have increased over the last several decades in China [1-2]. The one-month fatality of ICH is approximately 40% in survivors [3]. The one-year mortality of ICH patients is 58%, and 2/3 of survivors remain disabled [4]. Surgical evacuation for supratentorial hematoma is always controversial; some patients may benefit from it, but indications for surgical removal of the hematoma have not been conclusively defined [5]. In this case, we performed a direct puncture of the hematoma through the frontal burr hole. Then, we administered 15 min of massage by using multiple 1 mL quantities of normal saline and managed to reduce the volume of the hematoma as much as possible during the operation. For this patient, the result of our treatment, which omitted the standard craniectomy, was clearly successful. To our knowledge, this massage therapy for patients with ICH has not been previously reported in the literature.

## Case presentation

**Figure 1:**
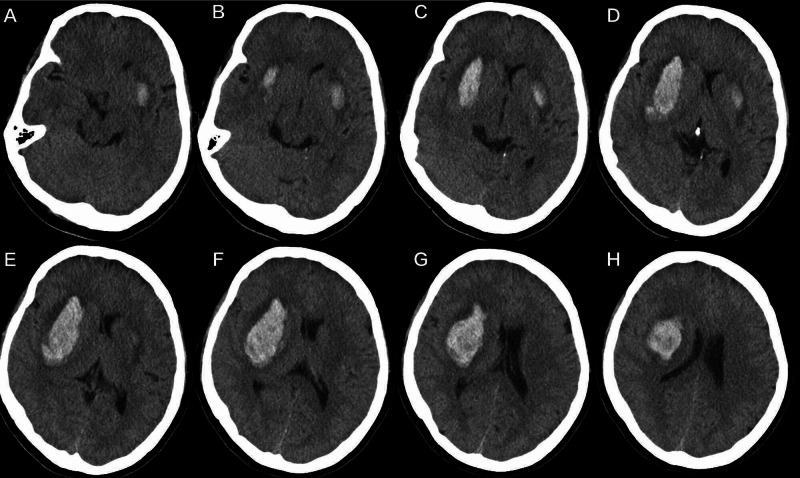
Preoperative axial CT of the patient. A-H: Preoperative axial CT of the patient. It shows bilateral intraparenchymal hemorrhages, mainly in the putaminal area.

A 48-year-old right-handed woman was brought to our hospital by her family, who stated that the woman could not speak well and had developed sudden left-sided weakness three hours previously. She had had longstanding hypertension and had never taken any antihypertensive drugs. The family denied any head trauma or seizures. No history of a previous stroke of any kind was obtained, and a review of her medications did not show any antiplatelet or anticoagulation administration. There was no family history of note. Her blood pressure was 185/105 mmHg with a regular pulse rate of 110 beats/min, a respiratory rate of 23 cycles/min, and a temperature of 37.5°C. She demonstrated flaccid, dense, left-sided weakness; extensor planting; and incomplete aphasia. An urgent noncontrast CT brain scan showed supratentorial bilateral intraparenchymal hemorrhages, mainly in the putaminal area (Figure 1).

**Figure 2:**
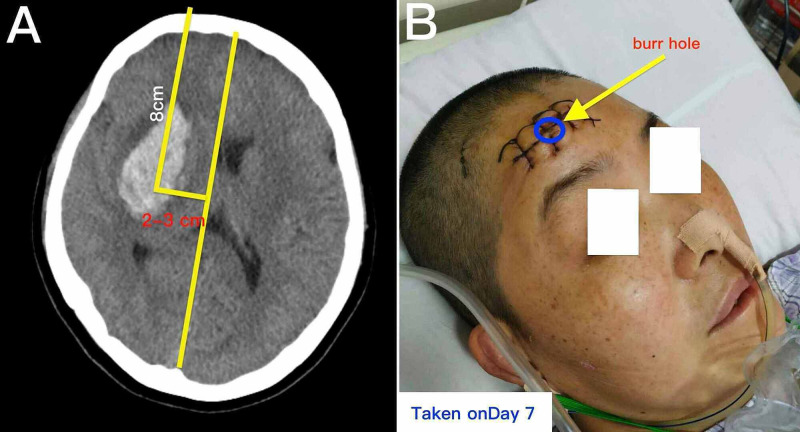
The site of the burr hole. A-B: The site of the burr hole. It was located 3 cm from the sagittal midline and 4 cm above the bottom slice that traverses the external auditory canal of the ear.

After consent by the patient's family, a standard burr-hole craniostomy was carried out. We selected the frontal area as the location of the burr hole (Figure 2). After inserting a catheter into the hematoma, we began to perform the maneuver of massage through the catheter by frequently using 2 mL quantities of normal saline. First, we injected 1 mL normal saline into the cavity of the hematoma and then aspirated 1 mL of the liquid out. Second, we injected the same 1 mL of normal saline into the hematoma and then suctioned it out again. When the liquid in the syringe became dull red, we then replaced it with another 2 mL of normal saline to repeat this maneuver. By performing this massage repeatedly, we progressively decreased the volume of the hematoma. After 15 min, we terminated the operation. The patient was admitted to the ICU, where she remained for three days. During this period, her blood pressure was controlled with amlodipine. Urokinase (40,000 U) was administered through the catheter every 12 hours for three days. There was no need for assisted ventilation. 

**Figure 3:**
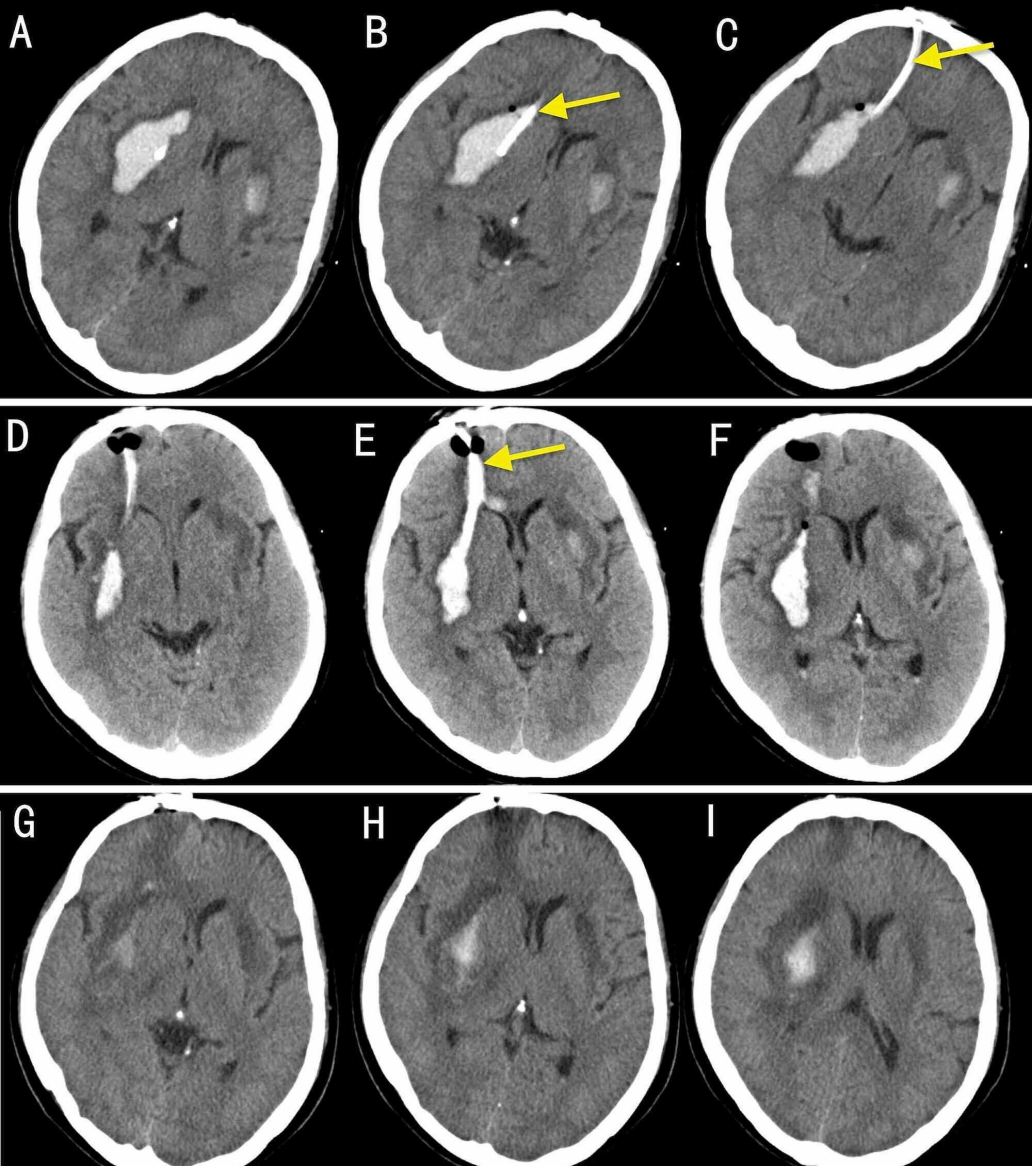
Postoperative axial CT images of the same patient. Postoperative axial CT images of the same patient. Upper row: Postoperative CT image (A, B, C) [Day 2] showing the catheter (yellow arrow) in the cavity. Middle row: D, E, F images [on Day 4] revealing that the residual hematoma had shrunk and showing the location of the catheter (yellow arrow). Lower row: H, I, G images [on Day 13] showing that the right-sided hematoma had almost disappeared and that the catheter had been pulled out.

The patient's neurological condition gradually improved, and on day 3, she was transferred to the general neurological ward. At that point, she was drowsy but able to answer simple questions and exhibited grade 1 left-sided pyramidal weakness, exaggerated reflexes, and extensor planting. The patient remained in the general ward for an additional 11 days and was then discharged home. At this point, she had grade 2 left-sided pyramidal weakness. Figure 3 shows the sequential development of the hematoma over 13 days. 

## Discussion

Intracerebral hemorrhage is usually defined as the abrupt onset of a focal neurological deficit caused by spontaneous hemorrhage into or around the brain. The craniotomy has been used over many years as treatment in routine practice, but large, pragmatic trials of this method have not shown improvements in these patients' prognosis [6-7].

Several preliminary studies revealed that minimally invasive surgery for ICH is safe and may improve functional outcomes [8-10]; however, the Minimally Invasive Surgery Plus rt-PA for Intracerebral Hemorrhage Evacuation (MISTIE III) trial found no clear benefit. Stemming from over 10 years of preliminary research, MISTIE III is actually an international, phase 3,500-patient clinical trial [11].

A meta-analysis was performed in 2008 and reviewed 10 randomized trials including 2059 patients [12]. The results showed that surgery was associated with a decreased risk of death and dependency (odds ratio: 0.71; 95% CI: 0.61-0.91). However, the benefit was not substantial, and there was remarkable heterogeneity for death as an outcome [12]. Recently, we have routinely performed natural drainage of ICHs rather than trying to aspirate certain blood clots during the operation. In this case, we performed persistent massage by using 1 mL quantities of normal saline and tried to reduce the volume of the hematoma as much as possible during the operation. Fifteen minutes of massage almost immediately decreased the volume of the hematoma. The modified Monro-Kellie doctrine tells us that a slight decrease in the volume of a hematoma will cause a dramatic improvement in the intracranial pressure (ICP) [13]. After thrombolytic evacuation of the clot with urokinase thrombolysis, our patient remained in the general ward for an additional 11 days and was then discharged home.

This is the first case to be best managed by minimally invasive surgery involving massage techniques and thrombolytic evacuation of an ICH. However, further studies need to be conducted to determine whether this method yields beneficial long-term outcomes compared to those of minimally invasive surgery alone.

In this report, a case of spontaneous ICH that was managed by minimally invasive surgery with massage techniques is presented. Fifteen minutes of massage administered during the operation almost immediately decreased the volume of the hematoma. We know that a slight decrease in the volume of a hematoma will cause a dramatic improvement in ICP. 

## Conclusions

Minimally invasive surgery involving massage techniques followed by thrombolytic evacuation of clots via urokinase thrombolysis will be a new method for treating ICH patients.

## References

[1] Guan T, Ma J, Li M (2017). Rapid transitions in the epidemiology of stroke and its risk factors in China from 2002 to 2013. Neurology.

[2] Zhou M, Wang H, Zhu J (2016). Cause‐specific mortality for 240 causes in China during 1990‐2013: a systematic subnational analysis for the global burden of disease study 2013. Lancet.

[3] van Asch CJ, Luitse MJ, Rinkel GJ, van der Tweel I, Algra A, Klijn CJ (2010). Incidence, case fatality, and functional outcome of intracerebral haemorrhage over time, according to age, sex, and ethnic origin: a systematic review and meta‐analysis. Lancet Neurol.

[4] Fogelholm R, Murros K, Rissanen A, Avikainen S (2005). Long term survival after primary intracerebral haemorrhage: a retrospective population based study. J Neurol Neurosurg Psychiatry.

[5] Hemphill JC 3rd, Greenberg SM, Anderson CS (2015). Guidelines for the management of spontaneous intracerebral hemorrhage: a guideline for healthcare professionals from the American Heart Association/American Stroke Association. Stroke.

[6] Mendelow AD, Gregson BA, Fernandes HM (2005). Early surgery versus initial conservative treatment in patients with spontaneous supratentorial intracerebral haematomas in the International Surgical Trial in Intracerebral Haemorrhage (STICH): a randomised trial. Lancet.

[7] Mendelow AD, Gregson BA, Rowan EN, Murray GD, Gholkar A, Mitchell PM (2013). Early surgery versus initial conservative treatment in patients with spontaneous supratentorial lobar intracerebral haematomas (STICH II): a randomised trial. Lancet.

[8] Hersh EH, Gologorsky Y, Chartrain AG (2018). Minimally invasive surgery for intracerebral hemorrhage. Curr Neurol Neurosci Rep.

[9] Hanley DF, Thompson RE, Muschelli J (2016). Safety and efficacy of minimally invasive surgery plus alteplase in intracerebral haemorrhage evacuation (MISTIE): a randomised, controlled, open-label, phase 2 trial. Lancet Neurol.

[10] Scaggiante J, Zhang X, Mocco J, Kellner CP (2018). Minimally invasive surgery for intracerebral hemorrhage. Stroke.

[11] Hanley DF, Thompson RE, Rosenblum M (2019). Efficacy and safety of minimally invasive surgery with thrombolysis in intracerebral haemorrhage evacuation (MISTIE III): a randomised, controlled, open-label, blinded endpoint phase 3 trial. Lancet North Am Ed.

[12] Prasad K, Mendelow AD, Gregson B (2008). Surgery for primary supratentorial intracerebral haemorrhage. Cochr Datab Syst Rev.

[13] Mokri B (2001). The Monro-Kellie hypothesis: applications in CSF volume depletion. Neurology.

